# Non-Invasive Microstructure and Morphology Investigation of the Mouse Lung: Qualitative Description and Quantitative Measurement

**DOI:** 10.1371/journal.pone.0017400

**Published:** 2011-02-25

**Authors:** Lu Zhang, Dongyue Li, Shuqian Luo

**Affiliations:** School of Biomedical Engineering, Capital Medical University, Beijing, China; Joint Research Centre - European Commission, Germany

## Abstract

**Background:**

Early detection of lung cancer is known to improve the chances of successful treatment. However, lungs are soft tissues with complex three-dimensional configuration. Conventional X-ray imaging is based purely on absorption resulting in very low contrast when imaging soft tissues without contrast agents. It is difficult to obtain adequate information of lung lesions from conventional X-ray imaging.

**Methods:**

In this study, a recently emerged imaging technique, in-line X-ray phase contrast imaging (IL-XPCI) was used. This powerful technique enabled high-resolution investigations of soft tissues without contrast agents. We applied IL-XPCI to observe the lungs in an intact mouse for the purpose of defining quantitatively the micro-structures in lung.

**Findings:**

The three-dimensional model of the lung was successfully established, which provided an excellent view of lung airways. We highlighted the use of IL-XPCI in the visualization and assessment of alveoli which had rarely been studied in three dimensions (3D). The precise view of individual alveolus was achieved. The morphological parameters, such as diameter and alveolar surface area were measured. These parameters were of great importance in the diagnosis of diseases related to alveolus and alveolar scar.

**Conclusion:**

Our results indicated that IL-XPCI had the ability to represent complex anatomical structures in lung. This offered a new perspective on the diagnosis of respiratory disease and may guide future work in the study of respiratory mechanism on the alveoli level.

## Introduction

Many lung diseases alter the morphology of the lung tissue [1]–[6]. Unfortunately, due to the limitation of resolution and contrast, at the early stage of the disease, minor pathological changes can not be discerned by conventional absorption-based imaging techniques. For example, lung cancer is one of the most common diseases with low survival rates worldwide; early detection of the lung cancer is known to improve the chances of successful treatment. But in most cases, cancer has been detected in the terminal stage and is impossible to be cured [1], [5], [7]. As a result, it is of great importance to image micro-structures in lung to enable early detection.

Histological biopsy is the most commonly used method for micro structures observation. But this is invasive and usually non-repeatable [8]–[10]. As one of the non-invasive methods, conventional radiography has been based purely on absorption contrast. However, the differences in X-ray absorption of biological soft tissues are quite small; this technique may lead to very low contrast and poor spatial resolution.

Phase contrast imaging (PCI) is a relatively new imaging technique, which can provide high contrast images by using phase shift of the X-ray. It is well known that X-ray is a form of electromagnetic wave. When it propagates through an object, both the amplitude and the phase of the wave are modified. It can be described by a complex reflective index [11], given by n = 1−δ−iβ. The imaginary part β is related to the attenuation based on absorption of the object, which we use in the conventional X-ray imaging. Unfortunately, the variations in X-ray absorption between different biological soft tissues are quite small; this technique may lead to very low contrast and poor spatial resolution. The real part δ is responsible for the X-ray phase shift. For biological soft tissues, the phase shift is almost one thousand times greater than the absorption term. Therefore, the aim of phase contrast imaging is to use the information of X-ray phase shift and convert it into image contrast [12]. This technique can greatly improve the image quality of soft tissues, particularly at the interface of tissues where the refractive index changes significantly. During recent years, it has been widely used by researchers for imaging of small animals. Among all the main PCI methods, in-line X-ray phase contrast imaging (IL-XPCI) is the simplest and the most straightforward, making it more suitable for clinical applications than other methods [13]. IL-XPCI has previously been used to study soft tissues of both human and small animals such as mice, rats, and rabbits. The results are satisfactory: high resolution images were obtained [14]–[16]. It may be an alternative method for lung observation and it is totally non-invasive.

To date, only a few studies have involved in-line X-ray phase contrast imaging to investigate lung of small animals. Due to the significant difference in density between air and tissues, the inner edges of the airway can result in marked edge enhancement in the phase contrast images. This has previously been proven by Suzuki et al [16]. Nevertheless, for the large number of alveoli, the fine structures were averaged out. Other researchers like Parsons and Sera et al. have utilized IL-XPCI to study lungs, and successfully generated the three-dimensional models of mouse airways [7], [17]. Despite advances in lung imaging using IL-XPCI, three-dimensional visualization of the alveoli in the intact animals has remained elusive. Nevertheless, the alveoli are important tiny structures through which gas exchange occurs. The morphology and distribution of alveoli can reveal information regarding lung health. For the diagnosis of drowning or other types of respiratory disorder like pneumonia, asthma, chronic bronchitis and emphysema, the shape of alveoli can be of considerable value [3], [18]. Furthermore, the morphology and growth of alveoli in embryos can reveal the secret of lung development, which has long fascinated biologists and mathematicians [19]. Therefore, the aim of this study is to explore these small alveoli.

In this study, IL-XPCI technique was used to image a 1-day-old mouse in situ. For the demonstration of complex microstructures, we combined IL-XPCI with computed tomography (CT) technique to provide three-dimensional images of an intact mouse lung. Moreover, we described our investigations on the anatomical structures of alveolar duct, alveolar sacs, and alveoli. Quantitative assessment was carried out on the morphology of the alveoli.

## Results

The lung is an essential respiration organ with complex three-dimensional configuration. About 90% of the lung is filled with air. IL-XPCI Computed tomography study was performed on a 1-day-old mouse. The results showed that the major features of the lung, including bronchi, bronchioles, and alveoli can be clearly displayed. Moreover, it is remarkable that the alveoli can be observed in three dimensions.

**Figure 1:**
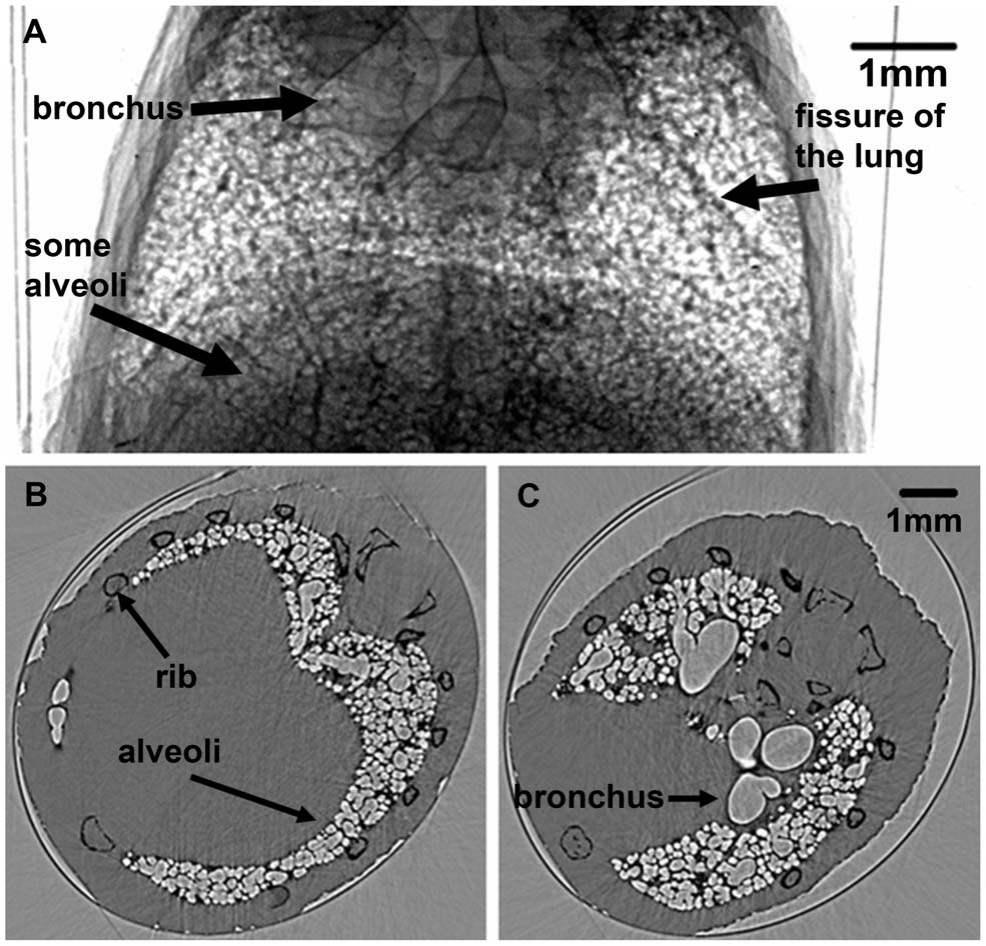
Images of the lung. One projection image is shown in (A). The bronchus and some alveoli are clear in this image. The dark line pointed by a black arrow is the fissure of the lung. (B) and (C) are two different CT slices. The bronchus and alveoli can easily be discerned in these images with an edge enhancement. The mouse rib, alveoli, and bronchus are indicated by the black arrows.

**Figure 2:**
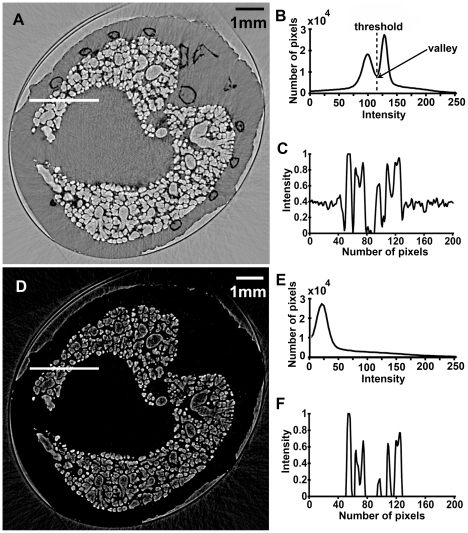
Comparison of the images between original and threshold-based segmentation result. (A) The original CT slice. (B) Histogram of the original CT slice. (C) Profile at the line drawn in (A). (D) CT slice after segmentation. (E) Histogram after segmentation and gray-scale transformation. (F) Profile at the line drawn in (D).

A CT projection image of the mouse lung is shown in Figure 1A. Although in this image, all the tissues overlapped with each other, some bronchi, lobes and many alveoli can be discerned. Figure 1B and C are two slices of the CT reconstruction images. Compared to conventional X-ray CT, in-line X-ray phase contrast CT had a much higher resolution (about 13 µm in our experiment). Bone, bronchi and alveoli were easily discernible with clear edges, which made it possible to separate different anatomical structures using the image segmentation technique. In order to extract the lung tissue and remove background noise, we applied a threshold-based image segmentation method to the CT slices. A comparison between original CT slice and threshold-based segmentation result is presented in Figure 2. The threshold was chosen at the valley of the histogram (Figure 2B). Other pixels with intensity lower than the threshold were set to zero. After gray scale transformation, the resulted image was shown in Figure 2D. From the profiles at the white line drawn in the same place of the two CT slices, the background noise in the image was considerably reduced.

**Figure 3:**
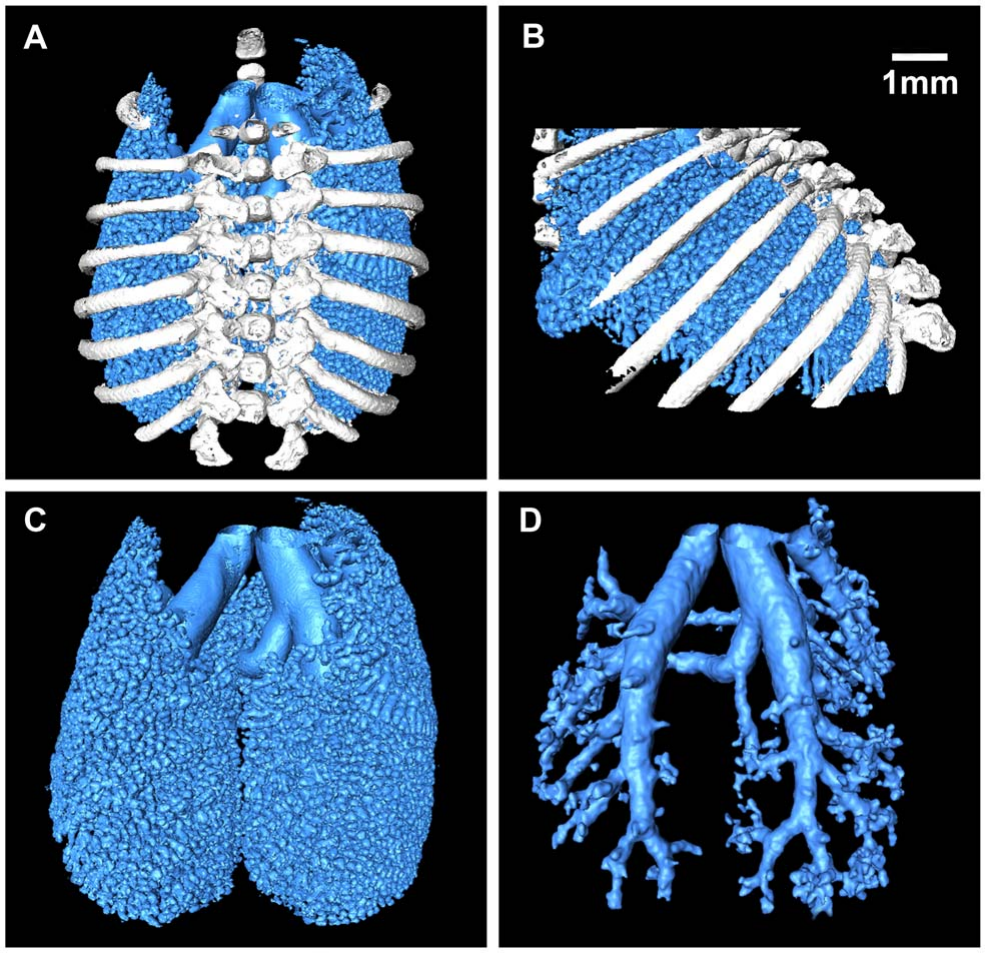
3D lung images. (A) Front view of the lung. (B) Right side view of the lung. (C) The bronchi and alveoli. See Video S1. We cut the 3D model in order to see the inner part of the lung. (D) The bronchial tree. The virtual endoscope video is also provided in the supporting information (Video S2).

Surface rendering method was used to visualize the lung, which allowed a clear depiction of complex spatial relationships and provided a strong space sense. By manually selecting the value of the reconstruction iso-surface, 3D models of tissues in the mouse chest cavity were obtained. All 3D models can be varied in size and rotated in real time to facilitate a detailed assessment of each structure (Video S1). As shown in Figure 3, a surface rendering view of lung is displayed. The three-dimensional relationships among bronchiole, alveolar duct and alveoli were all visible. Through the segmentation of the CT slices, three models were acquired. Figure 3A and B give an overview of chest which combines ribs, bronchi, bronchioles, and alveoli. Figure 3C focuses on the display of bronchi and alveoli. Figure 3D is the result of image segmentation; it highlights the bronchial tree. The virtual endoscope video is also provided in the supporting information (Video S2).

**Figure 4:**
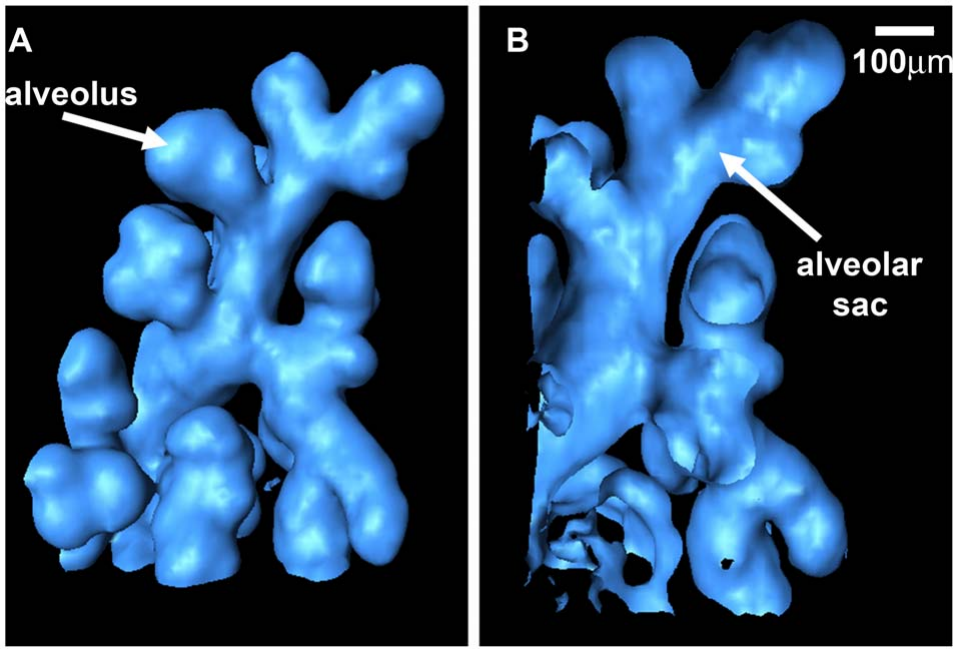
One small section of the lung. By the use of 3D image segmentation, this small section is separated from the lung tissue. (A) Front view. One alveolus is pointed by the white arrow. (B) Longitudinal section. A plane was used to cut the 3D model in order to show the inner part of the alveoli and alveolar scars, shown by the arrow.

Moving down the respiratory tract from the trachea, the air ducts divided more into smaller branches. The alveoli are the final branching of the respiratory tree, where the gas/blood exchange occurs. The distribution of the alveoli reveals information regarding lung health. Immature 1-day-old mouse alveoli range in diameter from 100 to 150 µm [15]. The diameter of most alveoli in our reconstruction image was in this range. Figure 4 is a detailed demonstration of one small section of the lung. These images provided a magnified view of the alveolar sacs and the alveoli. The model could be cut at any angle, varied in size, and rotated in real time. As shown in Figure 4, it was easy to measure the morphological characteristics, which was of great importance in the diagnosis of diseases related to alveolus and alveolar scar.

**Figure 5:**
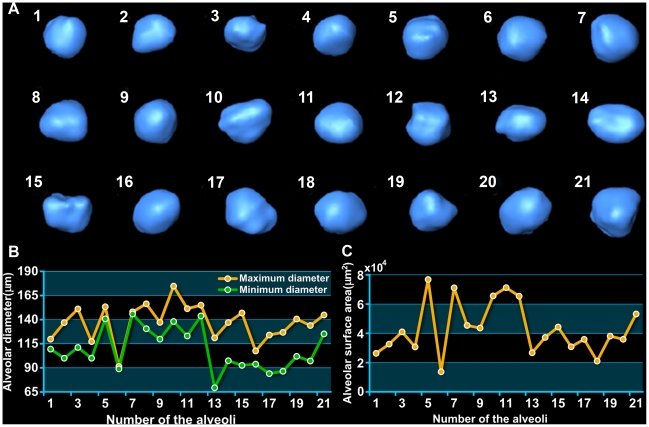
The 3D visualization and quantitative measurement of twenty-one single alveoli. (A) 3D reconstruction of the alveoli. They were chosen from different part of the lung randomly. (B) The maximum diameter and the minimum diameter of the alveoli. (C) The surface area of each alveolus.

In order to illustrate the alveoli more precisely, we only focused on several of them. Twenty-one single alveoli were chosen from different part of the lung. The three-dimensional images of the alveoli were shown in Figure 5A. The maximum diameter and the minimum diameter were measured in Figure 5B. The ratio of maximum and minimum diameters reflects the morphology of the alveoli to some extent. If this ratio approximates one, the shape of the alveolus is closer to a spheroid. The alveoli provide an enormous surface area for respiratory exchange; the change of alveolar surface area is closely related to lung health. For this reason, we calculated the surface area of each alveolus, as shown in Figure 5C.

## Discussion

The lungs are composed of sponge-like soft tissues. Detection of soft tissues using conventional absorption-based radiography is limited by the variation in tissue density. Since PCI is a phase-sensitive technique, it facilitates the visualization of fine structures, particularly in soft tissues. This technique has opened a new window for the visualization of lung. As a current imaging modality, clinical thin-section CT can be used to visualize airways with an internal diameter larger than 2 mm. It is better than conventional radiography and can provide more detailed information [6]. However, the terminal airspaces cannot be detected via this technique. Micro-CT for laboratory studies has a much higher resolution on the order of microns. Nevertheless, it is still based on the absorption information of the X-ray. The alveolar wall and the air space can not provide sufficient contrast to be observed [20]. A few studies have applied magnetic resonance imaging (MRI) to the lung imaging. Although MRI can provide excellent soft tissue imaging and functional imaging, the limitations lie in the low spatial resolution and long acquisition times [21]. The measurement of bronchial diameter will be hampered by these limitations, because normal spatial resolution of MRI is in the range of 4–6 mm and only a few can reach up to 1 mm [22]. The lung is an air-filled organ. At the air-tissue boundary, the refractive index changes significantly, so the lung becomes highly visible in PCI images. The lung images in our experiment have shown dramatic improvement in quality, and the alveoli can be clearly discerned. The theoretical spatial resolution of our experimental system is about 1 µm, while the diameter of mature mouse alveoli range from 38 to 80 µm and human alveoli are larger at 200–250 µm [15]. Thus, the resolution of PCI is adequate for micro-structures observation in lung. Though the radiation dose in our experiment was relatively larger than that used in clinical imaging, it can be reduced by the use of high energy X-ray, since contrast is not dependent on absorption of the beam [12], [15]. In addition, compared to other existing PCI methods, IL-XPCI requires relatively simple instrument. Most importantly, conventional laboratory X-ray source can be used as the light source, although longer exposure times are required [13]. The equipments are similar to conventional radiography. In this regard, it has the potential to be used in clinical diagnosis.

When it comes to studying the architecture of micro-structures in lung, histological biopsy is the most commonly used method [9]. But it is invasive and can not be repeated [10], [23]. During the process of invasive sampling, anesthesia is often required and tissue deformation frequently happen which is a major disadvantage when studying the morphology of the organs. There has long been a desire to explain breathing mechanism at the alveolar level. Unfortunately, a full understanding of these fine structures' original morphology is not available, because the lung will collapse when opening the thoracic cage at autopsy, due to the loss of negative pleural pressure [18]. Some researchers used confocal microscope to evaluate alveolar dynamics in the mouse lungs. However, this technique is limited by the depth of imaging (about 50 µm) [24]. How lungs develop has long fascinated biologists and mathematicians. Nevertheless, this process cannot be visualized in living embryos with current techniques [19]. Therefore, we adopted IL-XPCI to observe the lung non-invasively. An intact mouse was used to the maximum extent to preserve the organ's original morphology. In addition, previous studies have shown that deceased lungs can maintain a sufficient aeration level to be visualized [15].

In conventional radiography, it is impossible to conduct alveoli imaging without contrast agent. Although contrast agents are generally safe, adverse effects do sometimes occur [25]. In PCI experiment, some researchers instilled saline into the nasal airway of the mouse in order to see the alveoli [17]. Actually, the alveoli in human lungs had been visualized by conventional radiography. Some researchers used micro-CT to image human lungs [20]. In this case, an autopsy lung from a dead person was used and silver nitrate was needed as the contrast agent. However, in practice, when small pathological changes happen, it is impossible for doctors to open a live patient's chest or stain his/her alveoli to see where the lesion is. Therefore, non-invasive and non-staining technique is preferable.

In our study, the imaging of alveoli has shown promising results without any contrast agent. The 3D reconstruction technique provided an excellent view of lung and revealed structural details that were invisible to conventional radiography. The surface rendering results gave a perfect description of complex spatial relationships among bronchi, bronchioles and alveoli. The 3D volume data allowed virtual endoscope of the lung airways which helped the doctor visualize the 3D model of lung and perform a diagnosis without having to operate on the patient. Moreover, the measurement of alveoli showed the ability of PCI to observe the morphology of lung. Therefore, this technique has the potential use in disease diagnosis, such as asthma, chronic bronchitis and emphysema, which are associated with the size or morphology of lung airways. There are also potential uses in pharmacology when determining the optimum diameter of aerosolized drugs in lungs.

One limitation in our research was the study sample. Live animals were not imaged. Despite the advances in spatial resolution and contrast of phase contrast imaging, real-time X-ray phase tomography of the live animals has remained difficult. Live animals can only be imaged in two dimensions. The major obstacle in realizing this is the motion artifacts caused by breathing and cardiac motion of the animals, which will lead to serious blur in the reconstructed CT images. It is well known that the alveoli in lung are very small; any noise can influence the accuracy of the imaging result. Some attempts have been made to live animals imaging by taking the projection images at a specific breathing phase in synchrony with ECG signals [26]. But the animals have to be anaesthetized and with their breathing controlled by a ventilator. High-speed X-ray phase tomography may be another possible solution to live animal imaging by substantially reducing the imaging time. In fact, finishing a CT in one respiration phase is possible but the rotation speed of the sample stage and the time resolution of the detector need to be improved [27]. Another limitation was the lack of pathological samples. In this study, a normal 1-day-old mouse was used. The results proved that IL-XPCI is possible for lung micro-structures observation. Lung disorder models were missing, such as asthma, emphysema and lung cancer. We plan to evaluate these disorder models in our IL-XPCI imaging experiment as next steps, which are in progress now.

In this study, in-line X-ray phase contrast imaging was used to visualize a lung of an intact mouse. Our findings showed that IL-XPCI had the ability to represent complex anatomical structures in lungs. The three-dimensional model of lungs has been successfully established which provided an excellent view of lung airways. Thanks to the high resolution of the imaging, deep study was carried out on the tiny alveoli. A full understanding of the alveoli's architecture can facilitate the study of respiratory mechanism on the alveoli level. Single alveolus was displayed and measured. This offers a new perspective on the diagnosis of respiratory disease. Pathological changes can be detected by measuring the size of the lung airways, although further research and experimentation on lung is required to test this hypothesis.

## Materials and Methods

### Samples

All experiments and procedures carried out on the animals were approved by the animal welfare committee of Capital Medical University and the approval ID is SCXK-(Army) 2007-004. The study sample was a 1-day-old mouse, provided by Laboratory Animal Science, Capital Medical University. Before imaging, the mouse was humanely sacrificed by intraperitoneal (ip) sodium pentobarbital injection overdose. The mouse was imaged after four hours of its death in order to avoid the small shifts of the body caused by the development of rigor mortis [7]. And then a piece of Kapton (Dupont, DE, USA) was used and rolled up into a tube. This kind of film is an electrical insulation material with outstanding thermal, mechanical and chemical properties. The mouse was constrained in the tube, and placed on the sample stage in a vertically up-side-down position.

### In-line X-ray phase contrast imaging

The principle of IL-XPCI is based on Fresnel diffraction theory which can provide an edge-enhancement effect. Pogany, Gao and Wilkins first established the theoretical formalism for phase contrast image formation of weakly absorbing thin objects [28]. When X-ray beams travel through the object, the downstream beams carry the information of absorption and phase shift. The interaction between the object and the beam can be described by this transmission function
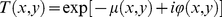
(1)where (x, y) is the spatial coordinates in the plane perpendicular to the propagation direction z, μ and ϕ are the attenuation and phase shift induced by the object. They are given by
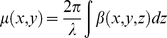
(2)

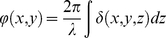
(3)where λ is the wavelength of the X-ray, β and δ are the imaginary and real parts of the refractive index, respectively. After propagating a sufficient distance, the phase shifts in the downstream beams are transformed into measurable intensity variations by means of Fresnel diffraction. An image detector records them as the phase contrast image. According to Fresnel diffraction theory and the wave front function, the Fourier transform of the recorded intensity can be approximated by
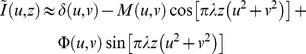
(4)where δ(u, v) is unit impulse function, (u, v) is the coordinate in Fourier domain, M and Φ are the Fourier transform of μ and ϕ respectively at the distance z. From Eq. (4) one can find that the recorded intensity of the image is determined by the phase shift and the absorption of the wave. Apparently, the optimal contrast depends on the spatial frequency, wavelength, and the object-detector distance [14].

**Figure 6:**
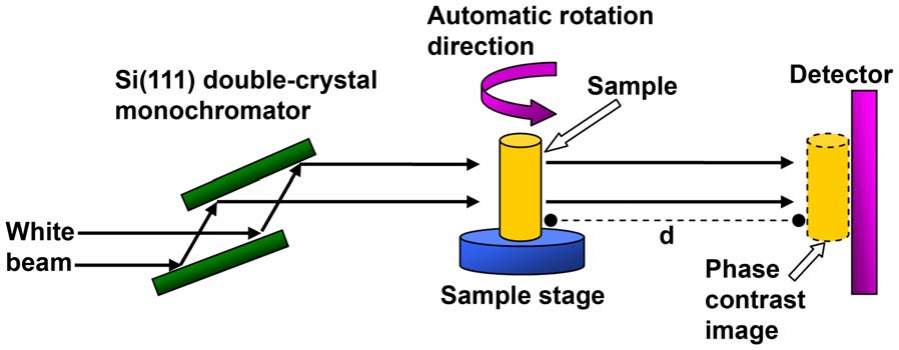
Schematic set-up of the IL-XPCI system. The mouse was constrained by a piece of Kapton film. During the CT imaging, the sample stage rotates 180° automatically. d is the propagation distance between the sample and the detector. In our experiment it was set to 1.2 m.

The in-line X-ray phase contrast imaging experiment was performed at X-ray imaging and biomedical application beamline (BL13W1) of Shanghai Synchrotron Radiation Facility (SSRF). SSRF is the third-generation synchrotron radiation source in China. Figure 6 shows the schematic image of the experiment setup. It consisted of two monochromator crystals, one automatic rotation sample stage and one X-ray sensitive CCD detector. The incident white synchrotron X-ray beam was first monochromatized by two Si (111) prefect crystals. The tunable energy range was from 8 to 72.5 keV, with the energy resolution of about 0.5%. In our experiment, it was adjusted to 18 keV. The theoretical spatial resolution of the system was about 1 µm. Subsequently, the highly parallel and monochromatic beam projected on the object was imaged. The CCD detector then recorded the transmitted beam at a distance of 1.2 m from the sample. During this distance, the downstream image was enhanced by Fresnel diffraction and the phase modulation was transformed via amplitude modulation. An X-ray sensitive CCD camera, which had maximum resolution of 4008 pixels×2672 pixels with 13 µm×13 µm each, was used as a two-dimensional detector to transform the beam into an image. During the CT data acquisition, the specimen was rotated around its cylinder axis for 180°. The number of projections was 1296, with exposure time of 80 milliseconds for each projection, and the total scanning time was 208 seconds. The surface dose was about 8 mGy for each projection. All the parameters were selected in order to obtain high quality images of the mouse lung.

### Image processing

Although IL-XPCI can provide high-resolution images of soft tissues, image processing is required during the analysis of the image. Two-dimensional projection images always suffer from spatial superimposition of the lesions; small lesions inside the tissue can not be detected. Therefore, 3D visualization method is preferable.

First of all, the background image was used for normalization of the projection images. For the reconstruction of the CT slices, conventional filtered back projection (FBP) algorithm was used [29].

The ribs are bone structures, which had a distinct gray scale in PCI reconstructed CT slices. Threshold-based method was used for the segmentation of ribs. Then an image segmentation method was applied to separate the lung tissues from the background. Since the histograms of our lung images had two peaks and a valley between them, the threshold for image segmentation can be chosen automatically at the bottom of this valley [30]. The other pixels with intensity lower than the threshold were set to zero. After this step, the ribs associated with many background signals considered as noise were removed. The whole lung airways can be segmented from this volume dataset by manually set a gray-level threshold. Due to the complexity of the lung structures and their irregular shapes and similar gray levels on the images, threshold-based image segmentation method was inadequate to separate the bronchial tree from the whole lung airways. In this study, we developed an image segmentation method based on 3D region growing to separate bronchial tree which was a semi-automatic procedure. It started in manually placing the seed point in the section of bronchus as well as setting the intensity threshold. Under the control of the intensity threshold, the growing would stop when the bronchial tree were separated.

After the above steps, three volume datasets of the ribs, the whole airways of the lung and the bronchial tree were obtained. Finally, the 3D models were generated by the use of surface rendering method. The surfaces are reconstructed by an iso-surface detection algorithm which allowed a clear visualization of the complex spatial relationships of anatomical features.

## Supporting Information

Video S1 **Animated view of rendered bronchi and alveoli.** This is the same 3D model shown in Figure 3C. The rotation of the model permits the viewers to observe the lung from different angles. The model can be cut from any angle in order to give a detailed view of the inner part of the lung. The bronchi, bronchioles, and alveoli are all visible.
(AVI)

Video S2 **The virtual endoscope from the bronchi to one alveolar duct.** This is the same model shown in Figure 3D. The volume data of the lung airways is separated from CT slices using 3D image segmentation method. 360° rotation of the model displays the spatial relationships of different lung airways. And the virtual endoscope of the model reveals the internal lung airways.
(AVI)
